# Supplementary material for: MethylSPWNet and MethylCapsNet: Biologically Motivated Organization of DNAm Neural Networks, Inspired by Capsule Networks
Source: NPJ Syst Biol Appl. 2021 Aug 20;7:33. doi: 10.1038/s41540-021-00193-7 (PMC8379254; doi:10.1038/s41540-021-00193-7)
Supplement: Supplementary file 1 — Supplementary Information [file 41540_2021_193_MOESM1_ESM.pdf]

Supplementary Material

Gene-Level CNS Tumor Embeddings

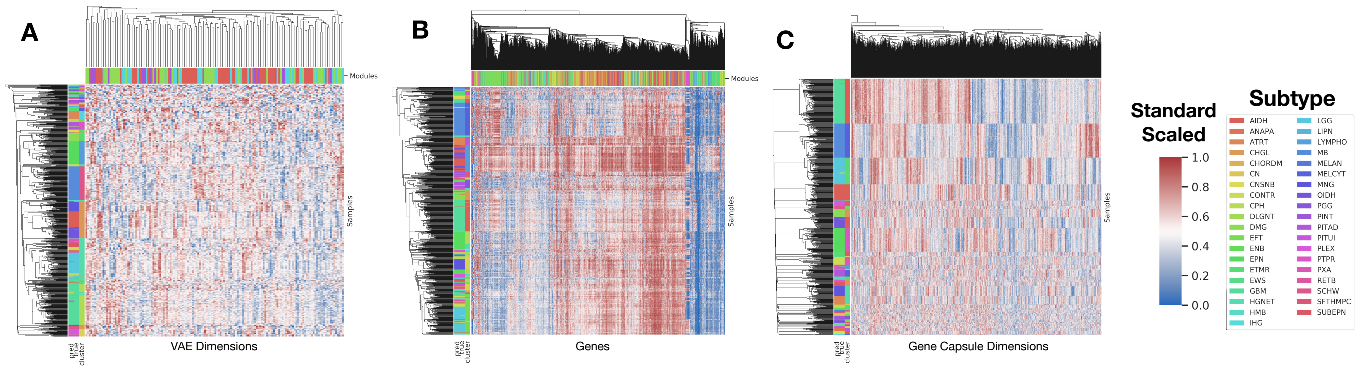

Supplementary Figure 1: Cluster heatmap plots of up to 2000 top components/capsules for: a) uninterpretable MethylNet dimensions, b) MethylSPWNet neural network defined gene methylation, c) 2200 dimensions comprising 55 MethylCapsNet capsules (55 capsules x 40 dimensions per Capsule = 2200 dimensions); samples constitute rows; capsules or latent dimensions constitute columns; rows are color labeled by predicted and true histological subtype and hierarchical clustering information; columns are color labeled by Louvain module assignment; all plots have been standard scaled to highlight patterns

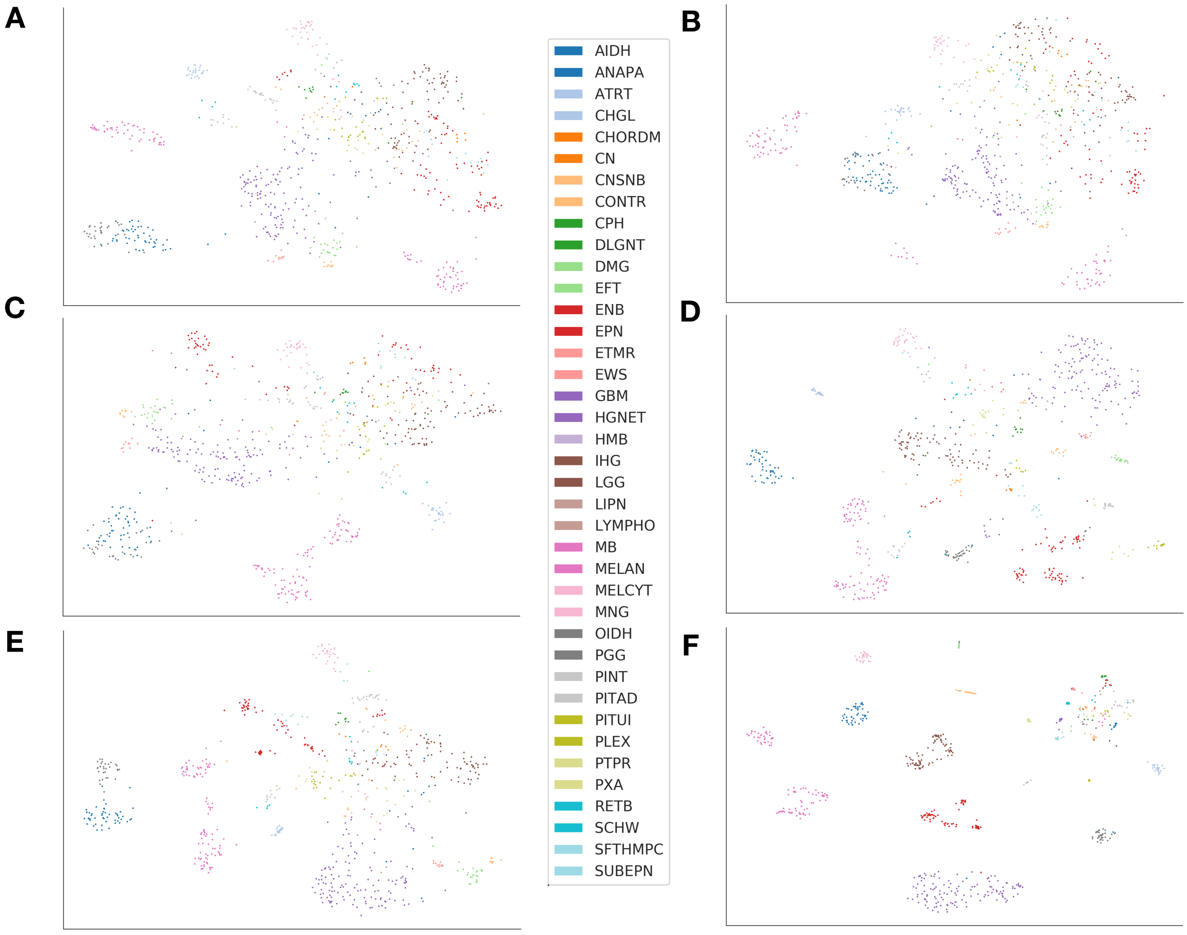

Supplementary Figure 2: NCVis Embeddings for a) all DNAm, b) Median gene DNAm, c) Median DNAm by gene context (upstream/downstream), d) *MethylNet*, e) *MethylSPWNet*, f) *MethylCapsNet*

Supplementary Table 1: Per-Subtype Breakdown of Classification Results for MethylSPWNet and MethylCapsNet on Test Set

	MethylSPWNet			MethylCapsNet			
**Subtype**	**Precision**	**Recall**	**F1-Score**	**Precision**	**Recall**	**F1-Score**	**Support**
**AIDH**	1.00	0.96	0.98	1.00	0.96	0.98	49
**ANAPA**	1.00	0.89	0.94	1.00	0.89	0.94	9
**ATRT**	1.00	1.00	1.00	1.00	1.00	1.00	24
**CHGL**	1.00	1.00	1.00	1.00	1.00	1.00	2
**CHORDM**	1.00	1.00	1.00	1.00	1.00	1.00	2
**CN**	1.00	1.00	1.00	1.00	1.00	1.00	5
**CNSNB**	1.00	1.00	1.00	1.00	1.00	1.00	9
**CONTR**	1.00	0.88	0.94	1.00	0.92	0.96	25
**CPH**	1.00	1.00	1.00	1.00	1.00	1.00	9
**DLGNT**	1.00	1.00	1.00	0.67	1.00	0.80	2
**DMG**	1.00	1.00	1.00	0.96	1.00	0.98	23
**EFT**	1.00	1.00	1.00	1.00	1.00	1.00	2
**ENB**	1.00	1.00	1.00	1.00	1.00	1.00	7
**EPN**	1.00	0.97	0.99	1.00	0.99	0.99	71
**ETMR**	0.91	1.00	0.95	0.91	1.00	0.95	10
**EWS**	1.00	1.00	1.00	1.00	1.00	1.00	3
**GBM**	0.98	1.00	0.99	0.98	0.99	0.98	130
**HGNET**	1.00	0.80	0.89	1.00	0.90	0.95	10
**HMB**	1.00	1.00	1.00	1.00	1.00	1.00	5
**IHG**	0.00	0.00	0.00	1.00	1.00	1.00	3
**LGG**	0.92	1.00	0.96	0.97	0.99	0.98	78
**LIPN**	1.00	1.00	1.00	1.00	1.00	1.00	2
**LYMPHO**	1.00	1.00	1.00	1.00	1.00	1.00	3
**MB**	1.00	1.00	1.00	1.00	1.00	1.00	100
**MELAN**	1.00	1.00	1.00	1.00	1.00	1.00	4
**MELCYT**	1.00	1.00	1.00	1.00	1.00	1.00	4
**MNG**	0.97	1.00	0.98	1.00	1.00	1.00	29
**OIDH**	0.94	1.00	0.97	0.94	1.00	0.97	31
**PGG**	1.00	1.00	1.00	1.00	1.00	1.00	4
**PINT**	1.00	0.90	0.95	0.91	1.00	0.95	10
**PITAD**	1.00	1.00	1.00	1.00	1.00	1.00	20
**PITUI**	1.00	1.00	1.00	1.00	1.00	1.00	6
**PLEX**	1.00	1.00	1.00	1.00	1.00	1.00	15

**PTPR**	1.00	1.00	1.00	1.00	1.00	1.00	6
**PXA**	1.00	0.92	0.96	0.92	0.85	0.88	13
**RETB**	0.80	1.00	0.89	1.00	0.75	0.86	4
**SCHW**	1.00	1.00	1.00	1.00	1.00	1.00	8
**SFTHMPC**	1.00	1.00	1.00	1.00	1.00	1.00	4
**SUBEPN**	1.00	1.00	1.00	1.00	1.00	1.00	13
**Overall Accuracy**			0.98			0.98	
**Macro-Average**	0.96	0.96	0.96	0.98	0.98	0.98	754
**Weighted Average**	0.98	0.98	0.98	0.98	0.98	0.98	754

Supplementary Table 2: Concordance Statistic from Cluster Assignment (k=14) to: cell of origin, molecular subspecification, and histology

Method	Cell of Origin	Histology+Molecular	Histology
**MethylNet**	0.72±0.012	0.68±0.0069	0.8±0.0097
**MethylSPWNet**	0.73±0.011	0.72±0.0059	0.81±0.0086
**MethylCapsNet**	0.65±0.012	0.66±0.0068	0.8±0.0078
**Median Methylation**	0.11±0.014	0.15±0.016	0.17±0.019
**Median Methylation by Context**	0.11±0.014	0.15±0.016	0.17±0.019

Preliminary Pathways and Module Analysis

Results (methods described in main text, section "Co-Methylation Embedding Modules") indicate that these top two modules are highly related to key cancer signaling pathways, including Rap1, Ras, WNT, and GABAergic pathways, to name a few. Alternative specification of key genes using the same module detection analysis for median methylation yielded no related pathways, whereas ranking genes by median absolute deviation recovered many pathways identified by *MethylSPWNet*, though not at the same power as that derived from the neural network. Furthermore, since the neural network pathway enrichment analysis discovered pathways with higher power than that specified using median methylation, it detected pathways with less well-known associations, such as Type I Diabetes Mellitus (hsa04940), corroborated by the literature [1]. Additionally, Type 1 diabetes is an autoimmune disease; we found associations with several immune evading markers, which may account for the spurious associations. All pathway enrichment tests are available in Supplementary Data 4.

Many similar, but not intersecting pathways were found to be statistically important for each of these analyses, as compared to the modularity-based analyses (methods described in main text, section "Analysis of CpG Weights derived from *MethylSPWNet*"). Particularly, some new pathways include axon guidance, Hippo signaling, Circadian entrainment, melanogenesis, TGF-B, and MAPK pathways. Complete results can be found in the additional spreadsheet. An analysis of the top 1000 hypervariable methylated CpGs across the cancer type yielded pathways that overlapped with *MethylSPWNet*

pathways, even though median absolute variation was not associated with the weight assigned to individual CpGs (Supplementary Figure 5; Supplementary Data 4).

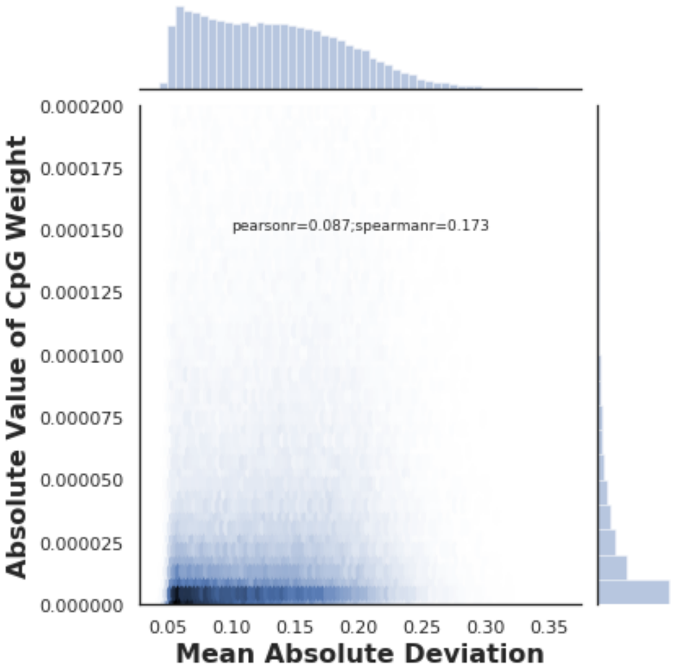

Supplementary Figure 3: Relationship of mean absolute deviation in DNAm across samples and CpG weight

Supplementary Table 3: Summarized results from preliminary pathway analyses (See supplementary data 4)

Summary of Notable KEGG Pathways	Notable Genes (n=9 sampled)	Average OncoScore	Gene Set Score
**Ras signaling pathway Homo sapiens hsa04014**	ABL1;PDGFRA;EGFR;EPHA2;ABL2;PRKACA;RALB;ETS2;PAK6	87.07	21.79
**Pathways in cancer Homo sapiens hsa05200**	ABL1;CCND1;ZBTB16;MECOM;PDGFRA;CDK6;PTEN;RUNX1;CCNA1	87.07	18.80
**Hippo signaling pathway Homo sapiens hsa04390**	LATS2;TP73;LIMD1;WWTR1;CTNNA1;LLGL2;DLG2;WNT5A;PARD3	82.93	31.10
**Axon guidance Homo sapiens hsa04360**	NTN1;EPHA2;UNC5A;EFNA1;PAK6;EPHB4;EPHA3;EPHB3;SLIT3	82.89	19.89
**Phospholipase D signaling pathway Homo sapiens hsa04072**	TSC2;EGFR;RALB;RHEB;MAPK1;GAB2;RALGDS;GNAS;PLD1	79.28	16.64
**Focal adhesion Homo sapiens hsa04510**	CCND1;PTEN;EGFR;ITGB4;MAPK1;VAV2;ITGB5;HGF;RAPGEF1	78.17	13.30
**MAPK signaling pathway Homo sapiens hsa04010**	MECOM;PDGFRA;EGFR;DAXX;MAP2K2;RPS6KA2;FGFR3;PRKCA;PDGFRB	76.62	20.23
**Gap junction Homo sapiens hsa04540**	PDGFRA;EGFR;MAP2K2;PRKCA;PDGFRB;PRKCB;GNAS;PDGFB;ADCY9	76.33	33.17
**Prostate cancer Homo sapiens hsa05215**	PDGFRA;EGFR;MAP2K2;CREB5;PDGFRB;CREBBP;TCF7L1;TCF7L2;PDGFB	76.33	27.64
**Melanoma Homo sapiens hsa05218**	PDGFRA;CDK6;EGFR;MITF;PDGFRB;FGF2;FGF1	76.33	26.57
**Calcium signaling pathway Homo sapiens hsa04020**	PDGFRA;EGFR;STIM1;PRKCA;PDGFRB;ITPKA;PRKCB;GNAS;CACNA1G	76.33	22.42
**Rap1 signaling pathway Homo sapiens hsa04015**	PDGFRA;EGFR;EPHA2;EFNA1;SIPA1;RALB;MAP2K2;BCAR1;MAPK1	76.33	20.27
**Type I diabetes mellitus Homo sapiens hsa04940**	TNF;PRF1;CD28;IL1A;LTA;ICA1;HLA-DRB5;HLA-DMA;HLA-C	73.23	30.35
**Adherens junction Homo sapiens hsa04520**	EGFR;CTNNA1;PTPN6;CREBBP;PTPRF;PARD3;TCF7L1;SMAD3;TCF7L2	69.06	69.62
**Estrogen signaling pathway Homo sapiens hsa04915**	EGFR;MAP2K2;CREB5;GNAS;HSPA1L;ADCY9;ADCY4;FKBP5;GABBR1	69.06	24.66

**Vascular smooth muscle contraction Homo sapiens hsa04270**	MYH11;MAP2K2;PRKCB;GNAS;ADCY9;ADCY4;PPP1R14A;PRKG1;ADRA1D	66.53	22.45
**Arrhythmogenic right ventricular cardiomyopathy (ARVC) Homo sapiens hsa05412**	CTNNA1;ITGB4;ITGA11;TCF7L1;ITGB7;TCF7L2;CTNNA2;ITGA8;SLC8A1	64.02	26.65
**Cell adhesion molecules (CAMs) Homo sapiens hsa04514**	CADM1;NCAM1;CDH3;PTPRF;CLDN9;CDH4;ITGB7;CADM3;ITGA8	60.56	17.10
**Glutamatergic synapse Homo sapiens hsa04724**	GLS2;PRKACA;MAPK1;PRKCA;PRKCB;GNAS;GNG7;GNG12;PLD1	56.32	22.85
**GABAergic synapse Homo sapiens hsa04727**	GLS2;PRKACA;GNG7;GNG12;GABRP;GNB1;GNG3;PRKCG;ADCY5	56.32	19.37
**Serotonergic synapse Homo sapiens hsa04726**	PRKACA;MAPK1;GNAS;GNG7;GNG12;GNB1;ALOX12B;PLA2G4B;GNG3	55.18	25.83
**Morphine addiction Homo sapiens hsa05032**	PRKACA;GNAS;GNG7;GNG12;GABRP;GNB1;GNG3;PRKCG;OPRM1	55.18	17.89
**Retrograde endocannabinoid signaling Homo sapiens hsa04723**	PRKACA;MAPK1;GNG7;GNG12;GABRP;GNB1;GNG3;PRKCG;ADCY5	55.18	17.66
**Melanogenesis Homo sapiens hsa04916**	MAP2K2;MITF;PRKCA;PRKCB;GNAS;CREBBP;TCF7L1;FZD6;TCF7L2	52.87	30.49
**Wnt signaling pathway Homo sapiens hsa04310**	CACYBP;PRKCA;PRKCB;CREBBP;TCF7L1;FZD6;SMAD3;TCF7L2;PRICKLE2	50.38	23.72
**Dilated cardiomyopathy Homo sapiens hsa05414**	ITGB4;GNAS;ITGA11;ITGB7;TGFB2;ADCY2;ADCY9;ADCY8;ADCY4	50.36	20.14
**Aldosterone synthesis and secretion Homo sapiens hsa04925**	CREB5;PRKCA;PRKCB;GNAS;CACNA1G;ADCY2;NR4A2;CAMK1D;CALML5	48.99	34.21
**Circadian entrainment Homo sapiens hsa04713**	PRKCA;PRKCB;GNAS;CACNA1G;ADCY2;GNB1;CALML5;ADCY9;ADCY8	46.73	29.09
**TGF-beta signaling pathway Homo sapiens hsa04350**	CREBBP;TGIF1;ACVR1C;SMAD3;SMAD7;TGFB2;INHBB;BMP7;PITX2	39.35	26.16

Context Enrichment Tables and Figures

We sought to evaluate genomic contexts independently that bring about patient phenotype. CpGs with the most positive neural network weights were found to be enriched for CpG islands (OR = 1.53; p-value<0.01), south shelves (OR=1.52; p=0.04), open sea regions (OR=1.18; p<0.01), 5'UTR regions (OR=1.52; p=0.05), and the first exon (OR=1.62; p=0.02). Positive weight CpGs were found to be depleted in north shores (OR=0.54; p<0.01), south shores (OR=0.63; p=0.05), gene bodies (OR=0.75; p=0.05) and TSS1500 (OR=0.72; p=0.02). Negative neural network weight CpGs were found to be enriched in north shelves (OR=2.47; p=0.05), north shores (OR=1.23; p=0.01), TSS200 (OR=1.24; p=0.05) and 5'UTR (OR=1.26; p=0.01). These negative weight CpGs were depleted in south shelf regions (OR=0.49; p=0.02), south shores (OR=0.81; p=0.04) and gene bodies (OR=0.86; p=0.02).

Supplementary Table 4: Fischer's Exact tests for enrichment of positive neural network weights for particular contexts

	**Number of Highest Positive Weighted CpGs**					
	**250**		**500**		**2100**	
	Odds Ratio	P-Value	Odds Ratio	P-Value	Odds Ratio	P-Value
**Island**	**1.53**	**0.00**	1.05	0.64	0.95	0.29
**N_Shore**	**0.54**	**0.00**	0.96	0.80	0.94	0.38
**N_Shelf**	1.49	0.11	1.03	0.84	0.85	0.14
**S_Shore**	**0.63**	**0.05**	0.76	0.08	0.92	0.26
**S_Shelf**	1.23	0.42	**1.52**	**0.04**	0.86	0.24
**OpenSea**	0.93	0.60	1.00	1.00	**1.18**	**0.00**
	**Number of Highest Negative Weighted CpGs**					
	**50**		**500**		**1200**	
	Odds Ratio	P-Value	Odds Ratio	P-Value	Odds Ratio	P-Value
**Island**	0.72	0.42	1.14	0.20	0.99	0.95
**N_Shore**	1.18	0.70	0.93	0.62	**1.23**	**0.01**

N_Shelf	**2.47**	**0.05**	0.91	0.76	0.81	0.16
S_Shore	0.90	1.00	1.04	0.77	**0.81**	**0.04**
S_Shelf	2.13	0.13	**0.49**	**0.02**	**0.68**	**0.03**
OpenSea	0.76	0.39	1.02	0.85	1.05	0.38

	Number of Highest Positive Weighted CpGs					
	150		200		500	
	Odds Ratio	P-Value	Odds Ratio	P-Value	Odds Ratio	P-Value
TSS1500	0.64	0.09	0.77	0.24	**0.72**	**0.02**
TSS200	1.21	0.46	1.21	0.44	1.07	0.63
5'UTR	**1.52**	**0.05**	**1.42**	**0.05**	1.07	0.61
1stExon	1.46	0.11	**1.62**	**0.02**	1.20	0.19
Body	0.79	0.16	**0.75**	**0.05**	1.03	0.75
3'UTR	0.85	0.85	0.63	0.31	0.98	1.00

	Number of Highest Negative Weighted CpGs	
	1000	
	Odds Ratio	P-Value
TSS1500	0.93	0.45
TSS200	**1.24**	**0.05**
5'UTR	**1.26**	**0.01**
1stExon	0.91	0.40
Body	**0.86**	**0.02**
3'UTR	1.17	0.29

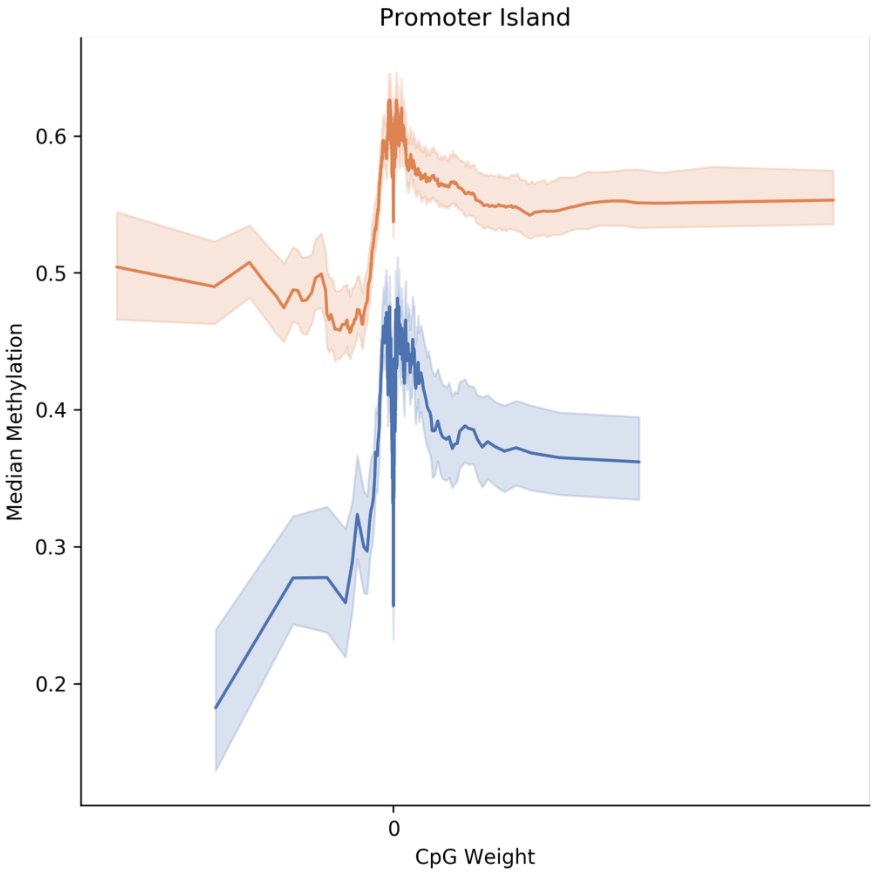

Supplementary Figure 4: Median methylation vs CpG weight; stratified by CpGs associated with island promoter (orange) and sites that are not associated with island promoters (blue); non-parametric bootstrapping was conducted across median aggregates for each tumor subtype to derive confidence intervals; note strong dip in median methylation near CpG weight of 0

Screenshot of Web Application and Network Plots

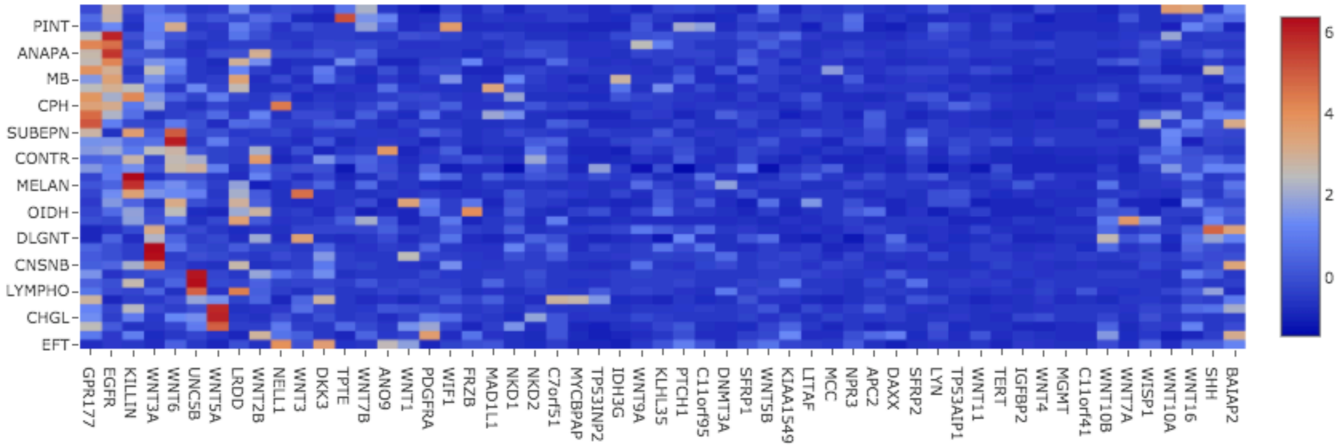

Supplementary Figure 5: Bipartite routing matrix that relates primary gene capsules to disease subtypes; red values indicate strong positive associations

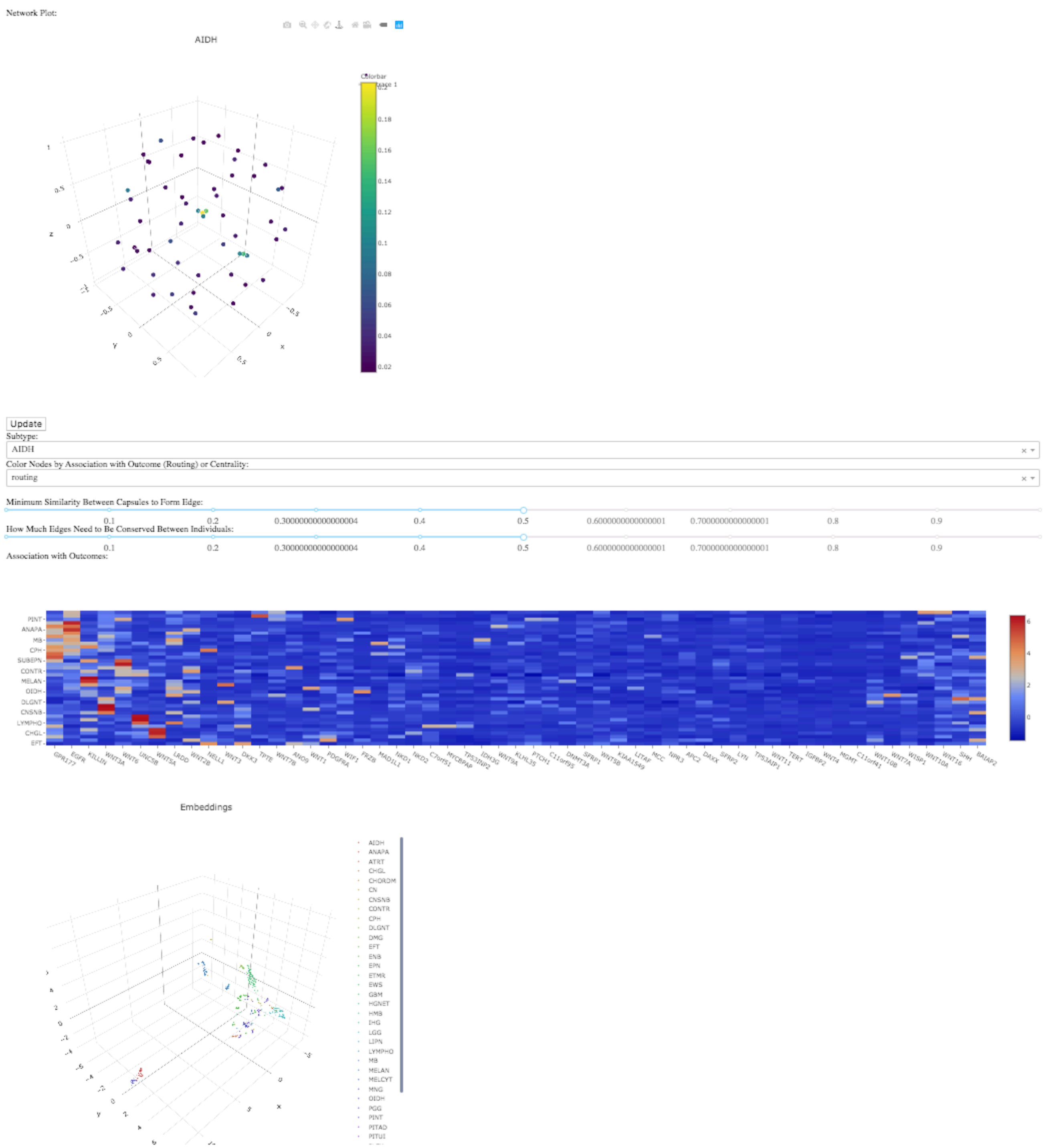

Supplementary Figure 6: Screenshot of entire web application interface where results are uploaded to; comprised of network discovery, routing matrix and embedding profile components

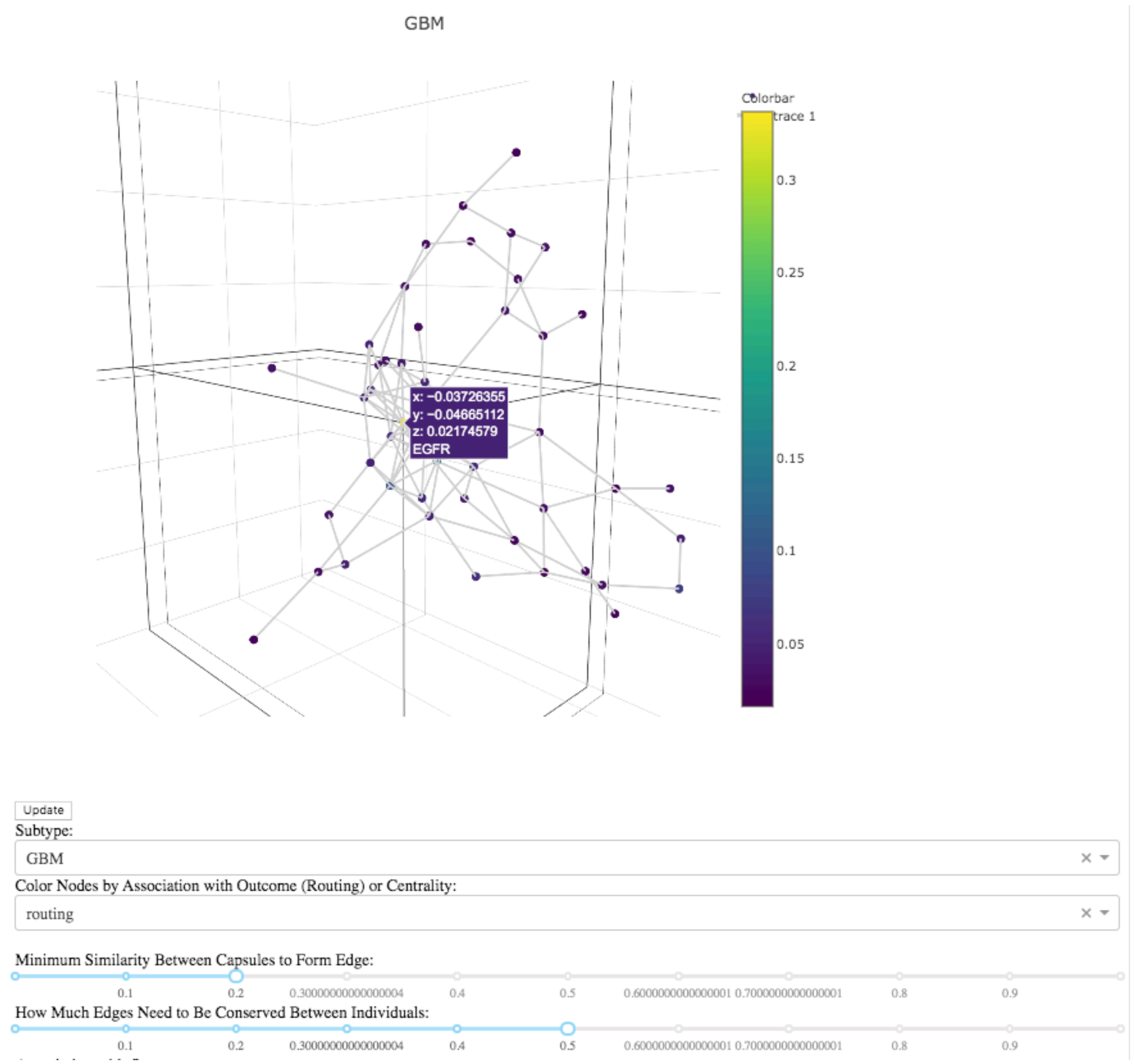

Supplementary Figure 7: Web application forms network using MethylCapsNet for GBM and uncovers EGFR as the most related gene using via the gene’s routing coefficient

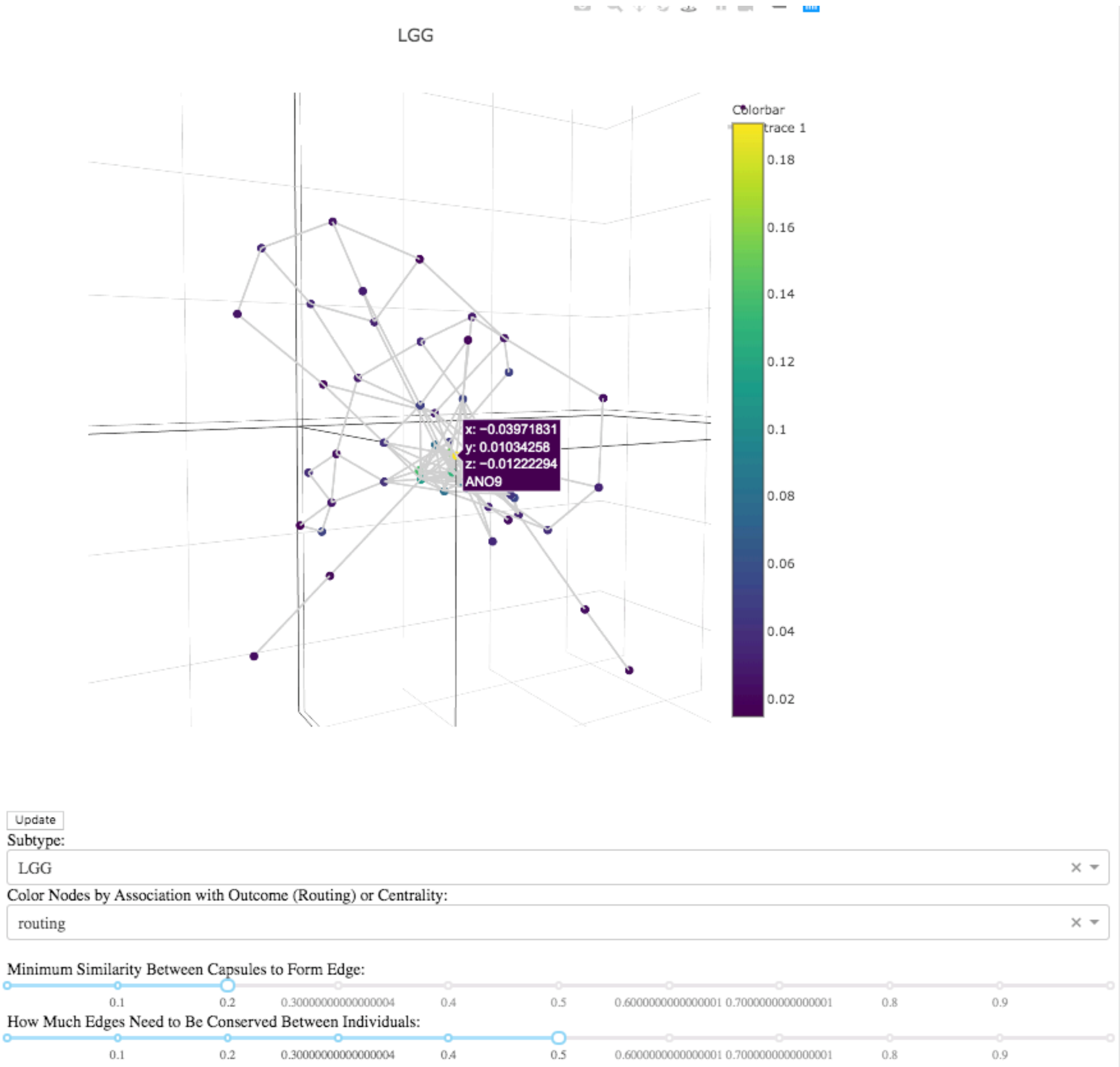

Supplementary Figure 8: Web application forms network using MethylCapsNet for LGG and uncovers ANO9 as the most related gene using via the gene's routing coefficient; previously implicated in [2] and related to the EGFR pathway

Alternative Capsule Formations and Cancer Subtypes

Here, we report classification results for an alternative set of datasets and capsules, as detailed in the results section: "Exploration of Alternative Capsule Formations and Cancer Subtypes". *Half Genes* are intersected with the following set of genes: https://github.com/Christensen-Lab-Dartmouth/MethylCapsNet/blob/master/test_data/brain_cancer_gene_list.txt . *New Genes* does not intersect with any of these genes. *Curated Genes* intersects with the following set of genes: https://github.com/Christensen-Lab-Dartmouth/MethylCapsNet/blob/master/test_data/breast_cancer_gene_list.txt

Supplementary Table 5: Classification performance for alternative selection of capsules; classification performance is macro-averaged and 95% confidence intervals estimated using 1000-sample non-parametric bootstrap

Dataset	Capsule Type	Algorithm	Accuracy	Precision	Recall	F1-Score
**CNS Tumor**	Half Genes	MethylCapsNet	0.98±0.0045	0.98±0.01	0.98±0.011	0.98±0.011
	New Genes	MethylCapsNet	0.98±0.0048	0.98±0.013	0.97±0.013	0.97±0.013
	Genomic Binned	MethylCapsNet	0.99±0.0043	0.99±0.01	0.97±0.012	0.98±0.011
**Breast Cancer**	Curated Genes	MethylCapsNet	0.79±0.029	0.84±0.035	0.71±0.035	0.75±0.036
	Genomic Binned	MethylCapsNet	0.75±0.031	0.74±0.035	0.75±0.036	0.74±0.034
	Island Promoters	MethylSPWNet	0.75±0.031	0.78±0.039	0.7±0.035	0.72±0.037
	N/A	MethylNet	0.77±0.03	0.75±0.033	0.78±0.033	0.76±0.032

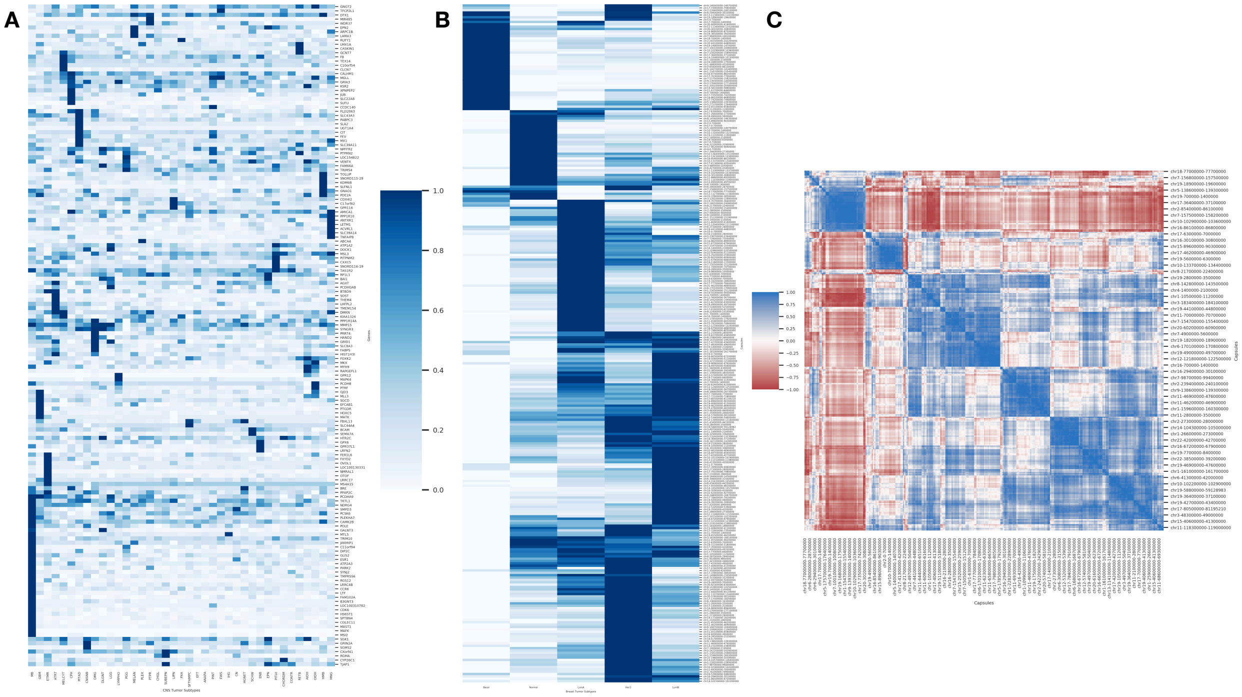

Supplementary Figure 9: Heatmaps of routing matrices (a,b; rows are capsules, columns are tumor subtypes) and unipartite projection of routing matrix (c; rows and columns are capsules) for the following scenarios: a) *Half Genes* for CNS tumor classification, b-c) 700kb non-overlapping *Genomic Regions* for PAM50 molecular classification

Supplementary Table 6: Set of genes utilized by MethylCapsNet for CNS classification; first set of genes reference set used for original classification task in paper; second two sets reference alternative sets of capsules that were formed; set of genes were selected from larger list of genes, but only included if they each have at least 10 annotated CpGs; gene list selection was done through inclusion/exclusion of a larger list of genes (eg. all/half/none found here: https://github.com/Christensen-Lab-Dartmouth/MethylCapsNet/blob/master/test_data/brain_cancer_gene_list.txt) and all genes

MethylCapsNet Original Genes

IDH3G	WNT7A	EGFR	LRDD	WNT1
WNT3A	WNT5A	SHH	NELL1	WIF1
GPR177	PDGFRA	C7orf51	ANO9	NKD1
WNT2B	SFRP2	WNT16	KLHL35	LITAF
WNT4	NPR3	SFRP1	DKK3	BAIAP2
WNT9A	TERT	WISP1	TP53AIP1	WNT3
WNT6	MCC	LYN	C11orf95	MYCBPAP
DNMT3A	NKD2	PTCH1	WNT11	APC2
WNT10A	DAXX	MGMT	C11orf41	TP53INP2
FRZB	KIAA1549	KILLIN	WNT10B	TPTE
IGFBP2	MAD1L1	UNC5B	WNT5B	WNT7B

MethylCapsNet Modified/Half Genes

MAP3K15	MEIS1	KIAA1549	DEAF1	BAIAP2
SYP	IGFBP2	GRM8	TP53AIP1	MGAT5B
FAM122C	CTDSPL	CAMK2B	USP2	TTYH2
IDH3G	NISCH	C7orf51	ELF5	FAM171A2
RNF207	WNT7A	GNA12	C11orf95	RICH2
DUSP27	WNT5A	LOC642006	WNT11	MPO
FGR	ZNF827	FASTK	C11orf92	KCNAB3
WNT3A	GPM6A	FOXP2	HOXC5	C17orf104
GPR177	NPFFR2	SFRP1	FBRSL1	MIR33B
WNT2B	C4orf42	KCNB2	DTX1	C17orf77
LGALS8	NPR3	WISP1	WNT5B	C19orf6
ALPL	TERT	DOCK5	WNT1	DLL3
C1orf61	MCC	NDRG1	CRIP2	PDE4A
WNT6	NKD2	ZMAT4	SLC7A8	MYH14
BIN1	SHROOM1	PAX5	C14orf23	HAS1
UXS1	LEMD2	MGMT	CGNL1	ZNF135
ASB18	PHACTR2	C10orf108	KIAA0182	RELB
WNT10A	C6orf25	UNC5B	NKD1	MMP9
NR4A2	UBD	PPYR1	SULT1A1	ZNF334
BOLL	BAT3	BLNK	LITAF	SCUBE1
STK25	TNF	EFCAB4A	HSF4	PIWIL3

MethylCapsNet Other/New Genes

HTR2C	JAKMIP1	CDK6	C11orf94	GLIS2
TKTL1	RGS12	MAFK	AMICA1	KDM6B
XPNPEP2	HAND2	FBXL13	SLC22A8	ATP2A3
F8	LETM1	ARPC1B	CIT	RAPGEFL1
GRIA3	NPFFR2	LRRC17	HOXC5	MSI2
MSL3	TMEM154	MLL3	KSR2	C17orf62
CXorf41	PCDHGA8	BAI1	DTX1	TEX14
KIAA1324	RUFY1	RP1L1	POLE	PTRF
SLFNL1	PCDHA9	SLC39A14	PITPNM2	GNGT2
LMX1A	LHFPL2	FER1L6	ACVRL1	GJD3
TAS1R2	LOC100310782	FAM66A	PABPC3	EPN2
GPR37L1	TNFAIP8	FABP5	PCDH8	FOXK2
ABCA4	CXXC5	EFCAB1	GPR12	SLC39A11
ATP1A2	SGCD	FAM102A	JUB	SOST
LOC100130331	GPX6	DOCK1	SLC8A3	MAPK4
THEM4	SLC44A4	DIP2C	MIR485	LAMA3
OTOF	TJAP1	VENTX	PTGDR	LRRC4B
FEV	PARK2	CALHM1	SNORD116-19	SPTBN4
BRE	PPP1R10	WDR37	SEMA7A	MAST1
FLJ32063	BTBD9	CYP26C1	SNORD115-29	B3GNT3
HS6ST1	TRIM10	GRID1	RGMA	MATK
ANTXR1	GPR116	MKX	PCSK6	PPAP2C
COLEC11	SYNJ2	SUFU	GRIN2A	BCAM
AGXT	ESR1	C10orf54	CLCN7	PPP1R14A
UGT1A4	SGK1	PDE2A	CASKIN1	DMKN
TFCP2L1	LRFN2	PLEKHA7	MMP15	SLA2
CCDC140	CCR6	SLC43A3	GNAO1	GCNT7
GALNT3	HIST1H3I	MTL5	NDRG4	COX4I2
TRIM54	PTPRN2	TOLLIP	NMRAL1	MX1
MGLL	CAMK2B	OVOL1	SYNGR3	TMPRSS6
LTF	LOC154822	FXYD2	SMPD3	MYH9
SGMS2	PRRT4	MS4A15		

Description of Supplementary Data

Appended to the supplementary material are a collection of files / supplementary data which demonstrate what kind of results to expect if running a differential embedding analysis, followed by a pathway and WGCNA analysis. The first tab of files 1-3 indicate results from an empirical bayes analysis. The second tab demonstrates pathway analyses of results from the first tab. Finally, tab 3 displays results from the WGCNA analysis. The first row indicates the module name, while the second row indicates the correlation with the subtype. The third row displays the significance of such association and remaining rows display top 20 hub genes for the WGCNA modules.

The fourth supplementary data file contains preliminary pathway results (see "Preliminary Pathways and Module Analysis"), where sets of genes were input into enrichr based on cutoff criteria discussed in the manuscript and supplementary material. The first tab displays pathways associated with genes that were produced by whether they contained some of the lowest CpG weights from MethylSPWNet. The second tab displays pathways for genes associated with the highest CpG weights from MethylSPWNet. The third tab displays pathways for genes that associated with hyper-variably methylated CpGs (as defined by median absolute deviation) across all cancer subtypes.

References

1. TONG, J. J., TAO, H., HUI, O. T. & JIAN, C. Diabetes mellitus and risk of brain tumors: A meta-analysis. *Exp Ther Med* **4**, 877–882 (2012).
2. Jun, I. *et al.* ANO9/TMEM16J promotes tumourigenesis via EGFR and is a novel therapeutic target for pancreatic cancer. *Br. J. Cancer* **117**, 1798–1809 (2017).
